# Low-Cost Vibrational Free Energies in Solid Solutions with Machine Learning Force Fields

**DOI:** 10.1021/acs.jpclett.3c03083

**Published:** 2023-12-15

**Authors:** Kasper Tolborg, Aron Walsh

**Affiliations:** † Department of Chemistry and Bioscience, Aalborg University, Fredrik Bajers Vej 7H, 9220 Aalborg Ø, Denmark; ‡ Department of Materials, Imperial College London, Exhibition Road, London SW7 2AZ, United Kingdom; § I-X, Imperial College London, Wood Lane, London W12 0BZ, United Kingdom; ∥ Department of Physics, Ewha Womans University, Seoul 03760, Korea

## Abstract

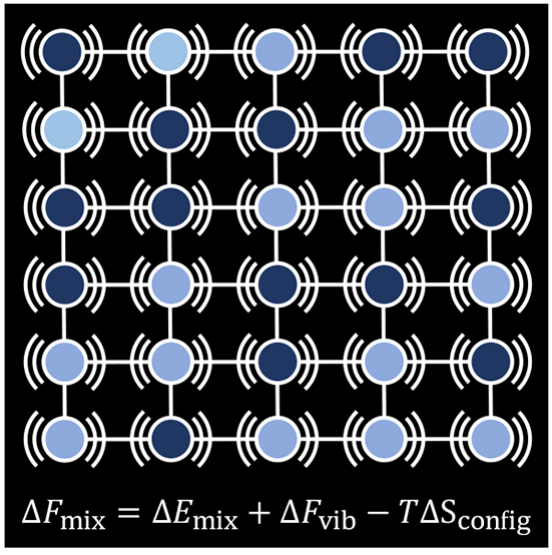

The rational design of alloys and solid solutions relies on accurate computational predictions of phase diagrams. The cluster expansion method has proven to be a valuable tool for studying disordered crystals. However, the effects of vibrational entropy are commonly neglected due to the computational cost. Here, we devise a method for including the vibrational free energy in cluster expansions with a low computational cost by fitting a machine learning force field (MLFF) to the relaxation trajectories available from cluster expansion construction. We demonstrate our method for two (pseudo)binary systems, Na_1–*x*_K_*x*_Cl and Ag_1–*x*_Pd_*x*_, for which accurate phonon dispersions and vibrational free energies are derived from the MLFF. For both systems, the inclusion of vibrational effects results in significantly better agreement with miscibility gaps in experimental phase diagrams. This methodology can allow routine inclusion of vibrational effects in calculated phase diagrams and thus more accurate predictions of properties and stability for mixtures of materials.

Alloys and solid solutions form an integral part of modern technology ranging from structural materials to semiconductors and catalysts. Recent years have seen renewed interest in compositionally complex materials with the advent of high-entropy alloys and compounds.^1−3^ First-principles calculations of compositional phase diagrams and derived properties can significantly aid the design and understanding of alloys and solid solutions.^2,4^ The most common tool for first-principles calculations of compositional phase diagrams is the cluster expansion (CE). In this method, the energy of an atomic configuration is expanded in a set of basis functions, and interaction parameters of pairs, triplets, etc., are fitted to total energies calculated with density functional theory (DFT).^4,5^ The energy, *E*, of a configuration, σ, is given as
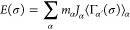
1where α is a cluster, *m*_α_ is the multiplicity of the cluster, Γ_α′_(σ) terms are the cluster functions constructed as a product basis fulfilling certain symmetry conditions, the angle brackets denote the average over symmetry equivalent clusters, and *J*_α_ terms are the effective cluster interactions (ECI), which are the parameters to be determined from fitting to DFT data.

In practice, the fitting of effective cluster interactions requires a range of structural relaxations with DFT of different (random or enumerated) configurations of the species forming the solid solution. From the determined ECIs, one can then predict the energy of any configuration, including those in supercells much larger than what can be simulated with DFT.

In the conventional approach to cluster expansion, it is assumed that configurational entropy is the main driving force for the formation of a solid solution. Thus, the internal energy of formation is used as the fitting target, and the configurational entropy is introduced through Monte Carlo sampling, from which the free energy of mixing can be extracted. In principle, there are several other contributions to the total free energy, of which vibrational entropy is often dominant^6,7^
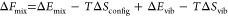
2

The vibrational contribution to the free energy is important for quantitative, or even qualitative, prediction of phase diagrams in several cases ranging from metallic alloys,^8−13^ ionic solid solutions,^14,15^ and covalent solid solutions^16^ to complex hybrid organic–inorganic materials^17^ and high-entropy alloys and compounds.^18−20^ The vibrational free energy can either stabilize or destabilize the formation of solid solutions depending on the sign of Δ*S*_vib_,^10^ and it may furthermore have effects on the short-range order and physical properties.

Assuming that the configurational and vibrational origins of entropy can be separated due to their different time scales, the vibrational entropy can be calculated for each configuration and introduced into the cluster expansion, by replacing the total (internal) energy, Δ*E*_mix_, in eq 1 with the free energy (Δ*F*_vib_ = Δ*E*_mix_ + Δ*E*_vib_ – *T*Δ*S*_vib_). The straightforward procedure would be to perform lattice dynamics calculations for each structure in the training set to determine the vibrational free energy within the harmonic phonon approximation. However, DFT-based lattice dynamics calculations require the calculation of large supercells and are thus usually not feasible for the hundreds of (low-symmetry) training structures often used for fitting cluster expansions. Thus, in practice, simple surrogate models based on, e.g., bond length versus bond strength relations or empirical potentials are often used to determine force constants for lattice dynamics calculations, even today despite the increases in computational power.^6,11^

Machine learning force fields (MLFFs) are evolving rapidly and have been successfully employed over the past decade to perform molecular dynamics (MD) simulations with near-DFT accuracy over time and length scales intractable for DFT-based MD.^21−24^ The force fields can be trained on various data generated from, e.g., hand-crafted structures, molecular dynamics simulations, or randomly generated configurations and often show remarkably good agreement with properties, including phonon dispersions, calculated from DFT.^25,26^ Recently, universal force fields have been developed and trained on data from the large database of geometry optimizations (energies, forces, and stresses) found in Materials Project.^27,28^ These have shown great promise for structural optimization, lattice dynamics calculations, and even MD simulations.

Inspired by the recent successes in training force fields on geometry relaxations, we here propose to build an MLFF trained on the relaxation trajectories already available when constructing a cluster expansion model. We then use this force field as a surrogate model for DFT to perform lattice dynamics calculations for the training structures to include vibrational free energies in the cluster expansion model with a low additional computational cost. In this Letter, we demonstrate this method for two compositionally simple materials, the Na_1–*x*_K_*x*_Cl (rock salt structure) and Ag_1–*x*_Pd_*x*_ (fcc structure) solid solutions as archetypical representatives for ionic and metallic solid solutions, respectively. For the former, previous simulations have shown that vibrational entropy is key to obtaining a miscibility gap in agreement with experimental results.^14^

*Force Field Construction and Performance.* We first demonstrate the methodology for the Na_1–*x*_K_*x*_Cl solid solution. MLFFs are constructed from the relaxation trajectories using a flavor of the Gaussian approximation potential (GAP) method with the smooth overlap of atomic positions (SOAP) radial and angular descriptor (see Computational Methods). The training error of the MLFF on forces from DFT is 4.8 meV Å^–1^ (all data), and the validation error evaluated on the last 31 rattled structures is 6.5 meV Å^–1^ (when 600 relaxation trajectories are included). The learning curve of the MLFF trained on relaxations included in the cluster expansion is shown in Figure S4, and parity plots are shown in Figure S5. After the inclusion of 150–250 relaxation trajectories in the training data, the validation error is already relatively low, though with an overall nonsmooth decrease.

The most important indicator of performance in this case is the ability to accurately predict the phonon frequencies and even more importantly the vibrational free energies derived thereof. Figure 1 and Figures S6 and S7 show comparisons of phonon dispersions, density of states (DOS), and vibrational free energies for a selected set of training structures, including the end-members, i.e., pure NaCl and KCl (Figure S6). It is evident that the MLFF reproduces the DFT phonon dispersions and DOS with overall good agreement, and in the temperature range of interest, the error in vibrational free energy is typically on the order of 1–2 meV atom^–1^. As DFT internal energies are commonly converged to ∼1 meV atom^–1^, this should be considered an acceptable error range. Note, however, that the errors are given per atom, whereas the relevant energies for the construction of CEs are given per metal atom (or formula unit), i.e., twice as large for Na_1–*x*_K_*x*_Cl.

**Figure 1:**
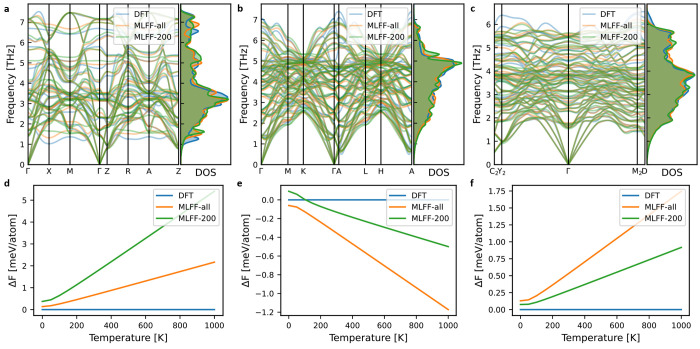
(a–c) Phonon dispersions and density of states (DOS) and (d–f) vibrational free energies of selected structures of Na_1–*x*_K_*x*_Cl from the training set compared with those calculated from DFT. Panels d–f show the difference in vibrational free energy with respect to the DFT results (blue line). MLFF-all indicates the force field trained on all relaxation data and is the one used for production simulations, and MLFF-200 indicates the one trained on 200 relaxation trajectories. The crystal structures and compositions of these selected example cases are shown in Figure S8.

The error in the vibrational free energy for the end-members, especially NaCl, is significantly larger than for the mixed systems (Figure S6). We attribute this to a poor cancellation of errors for systems with few atoms and thus few phonon branches, despite the seemingly similar agreement in terms of phonon dispersion and DOS. Because the vibrational free energy is calculated from a sum over the phonon frequencies, one can expect mixed systems with more phonon branches to have both overestimated and underestimated frequencies resulting in a cancellation of errors, which is less likely for the pure phases with few atoms. For this reason, we will construct two temperature-dependent cluster expansions: one with all vibrational free energies calculated with the MLFF and one with the vibrational free energies of the end-members calculated with DFT. Because calculating the end-member phonon dispersions with DFT is relatively cheap and should always be part of the validation of the MLFF, this comes at no additional computational cost.

Finally, we observe that the overall agreement with DFT is already good for the MLFF trained on a smaller data set, though with some significant outliers, especially for pure NaCl and the structure depicted in Figure 1d.

*Compositional Phase Diagram.* The compositional phase diagram is calculated using Monte Carlo simulations in the variance-constrained semigrand canonical (VCSGC) ensemble, which allows for efficient mapping of multiphase regions.^29,30^ Three CEs are constructed for the simulations: (i) a temperature-independent CE based on only internal energies from DFT, (ii) a temperature-dependent CE with all vibrational free energies calculated by the MLFF, and (iii) a temperature-dependent CE with the vibrational free energies of the end-members calculated from DFT and the remaining free energies calculated with the MLFF. In all cases, the cluster expansion is fitted to mixing (free) energies and constrained to reproduce the (free) energies of the end-members exactly.

A comparison of reference DFT (+ *F*_vib_) and CE data is shown for all three models in Figure S9. Their root-mean-square errors (RMSEs) from 10-fold cross-validation are 2.5, 3.7, and 4.1 meV per formula unit. While the RMSE is generally low, we note that there are some outliers, especially when the vibrational free energy is included, which is to be expected from the observed differences between DFT and MLFF vibrational free energies. To some degree, we expect that the CE can act as a noise filter due to the large amount of data present, at least in the absence of systematic errors. Generally, we observe that the mixed phases are significantly stabilized due to the vibrational free energy.

The calculated compositional phase diagram is shown in Figure 2 and compared with experimental results tabulated by Thompson and Waldbaum.^31^ The main feature of the phase diagram is a miscibility gap. Neglecting the vibrational free energy, as typically assumed for solid solutions studied with cluster expansion techniques, results in a miscibility gap extending to temperatures much higher than those experimentally observed, in agreement with previous DFT studies.^14^ The inclusion of vibrational effects lowers the miscibility gap significantly, to values in much better agreement with experiment. For the CE based on only MLFF vibrational free energies, the highest immiscibility temperature is calculated to be 950 K at *x* ≃ 0.4, close to the experimental value of 770 K.

**Figure 2:**
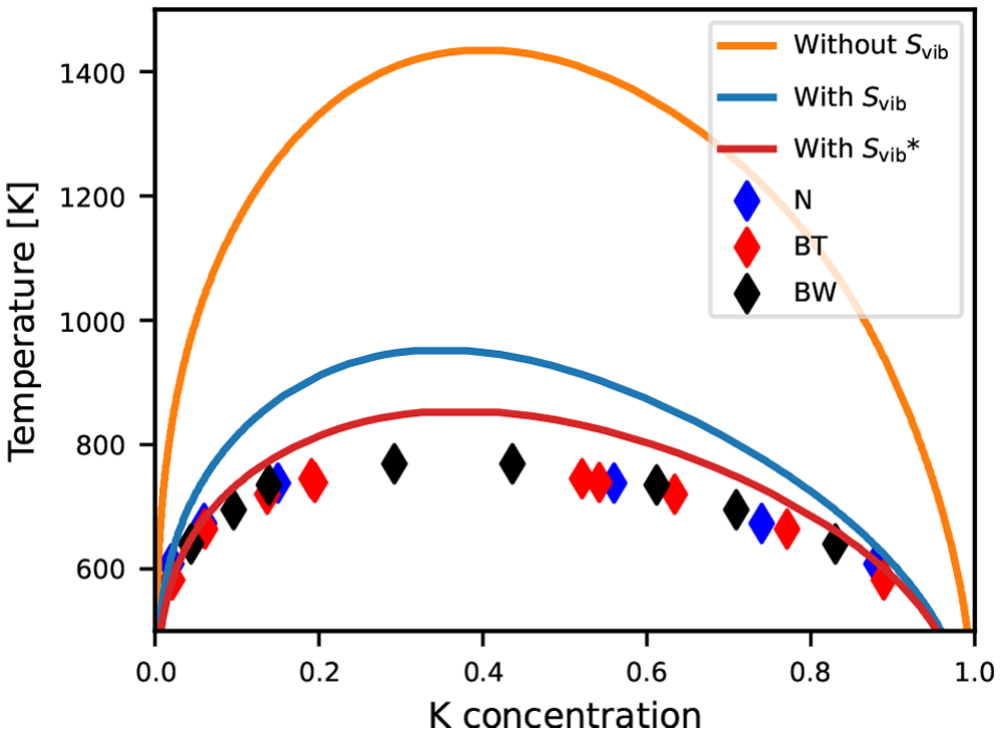
Compositional phase diagram of Na_1–*x*_K_*x*_Cl calculated from MC simulations in the VCSGC ensemble using cluster expansions with and without inclusion of the vibrational entropy. For those with the vibrational entropy included, the red curve marked by an asterisk is the one in which the vibrational free energies of the end-members are calculated with DFT. Experimental results from Nacken (N),^32^ Bunk and Tichelaar (BT),^33^ and Barrett and Wallace (BW)^34^ tabulated by Thompson and Waldbaum^31^ are given for comparison.

From Figure S6, we note that the vibrational free energies of the end-members, particularly for NaCl, are predicted to be too low compared to the DFT results, thus resulting in an overstabilization of the pure phases, which should lead to a less miscible solid solution. Constraining the end-member free energies to those calculated from DFT-based lattice dynamics, thus, leads to a further stabilization of the solid solution, with a maximum temperature of the miscibility gap of 850 K, which is <100 K above the experimental observation. Furthermore, we note that the asymmetry of the miscibility gap is well reproduced.

The overall agreement with experiment here is significantly better than that obtained from the bond length–bond strength relations applied by Burton and van de Walle.^14^ In particular, the asymmetry and behavior for more dilute solid solutions are better reproduced here.

To investigate the effect of the accuracy of the force field and of constraining the energy of the end-members, we provide two additional tests. (i) We use the force field trained on only the first 200 relaxation trajectories (denoted MLFF-200 in Figure 1), and (ii) we follow the same procedure as described above but do not enforce the cluster expansion to reproduce the (free) energies of the pure phases exactly. The cluster expansion fit and calculated phase diagram of (i) are shown in Figures S10 and S12, respectively. We note that the vibrational free energy of NaCl is poorly reproduced with MLFF-200 (Figure S6), and thus, relying on only the MLFF gives a poor phase diagram. However, using the vibrational free energy from DFT for the end-members results in a phase diagram in good agreement with experiment also for this force field, which is trained on significantly fewer data. Similarly, the cluster expansion fit and calculated phase diagram of test (ii) are shown in Figures S11 and S13, respectively. We note that this leads to large deviations in the (free) energy of the end-members between cluster expansion and reference energies, because the large amount of data for mixed phases biases the cluster expansion toward these. Generally, this leads to smaller mixing (free) energies (Figure S11) and results in a miscibility gap at lower temperatures (Figure S13) compared to the constrained model shown in Figure 2.

These different tests highlight the precision of the phase diagram reconstruction resulting from the different model choices, both in terms of conventional cluster expansion choices such as whether to constrain the model to end-members and in terms of the accuracy of the vibrational free energy. In all cases, the inclusion of vibrational entropy significantly improves the agreement with the experimental results.

*Extension toAg*_*1*–*x*_*Pd*_*x*_. For the Ag_1–*x*_Pd_*x*_ solid solution, an MLFF is trained in a way similar to that of Na_1–*x*_K_*x*_Cl. Figure S14 shows the training and validation errors as a function of training set size, and Figure S15 shows the parity plot. The final training error is 4.8 meV Å^–1^ (all data), and the validation error evaluated on the last 31 rattled structures is 17.2 meV Å^–1^ (when 600 relaxations are included). A trend similar to that of Na_1–*x*_K_*x*_Cl with a decrease in validation error with training set size and a flattening of the curve after ≃150 trajectories is observed, but we note that the validation error is significantly larger here.

The accuracy of the MLFF on phonon dispersions and vibrational free energies for a selected set of training structures are shown in Figure 3 and for pure Ag and Pd in Figure S16. The agreement with the DFT values is similar to that of Na_1–*x*_K_*x*_Cl. Again, we note that the error in vibrational free energy is larger for the end-members, although visually the phonon dispersions are in very good agreement. Thus, we will again simulate the phase diagram both with and without end-members constrained to DFT values. A comparison of DFT (+ *F*_vib_) and CE energies is shown in Figure S18, including their cross-validation RMSE. Compared to the Na_1–*x*_K_*x*_Cl case, a smaller effect of vibrational entropy and a different kind of stabilization is observed. In Ag_1–*x*_Pd_*x*_, the structures around *x* ≃ 0.5 are slightly destabilized relative to the end-members at increased temperatures, which results in a relative stabilization of mixed phases around *x* ≃ 0.8 as will be clear from the phase diagram.

**Figure 3:**
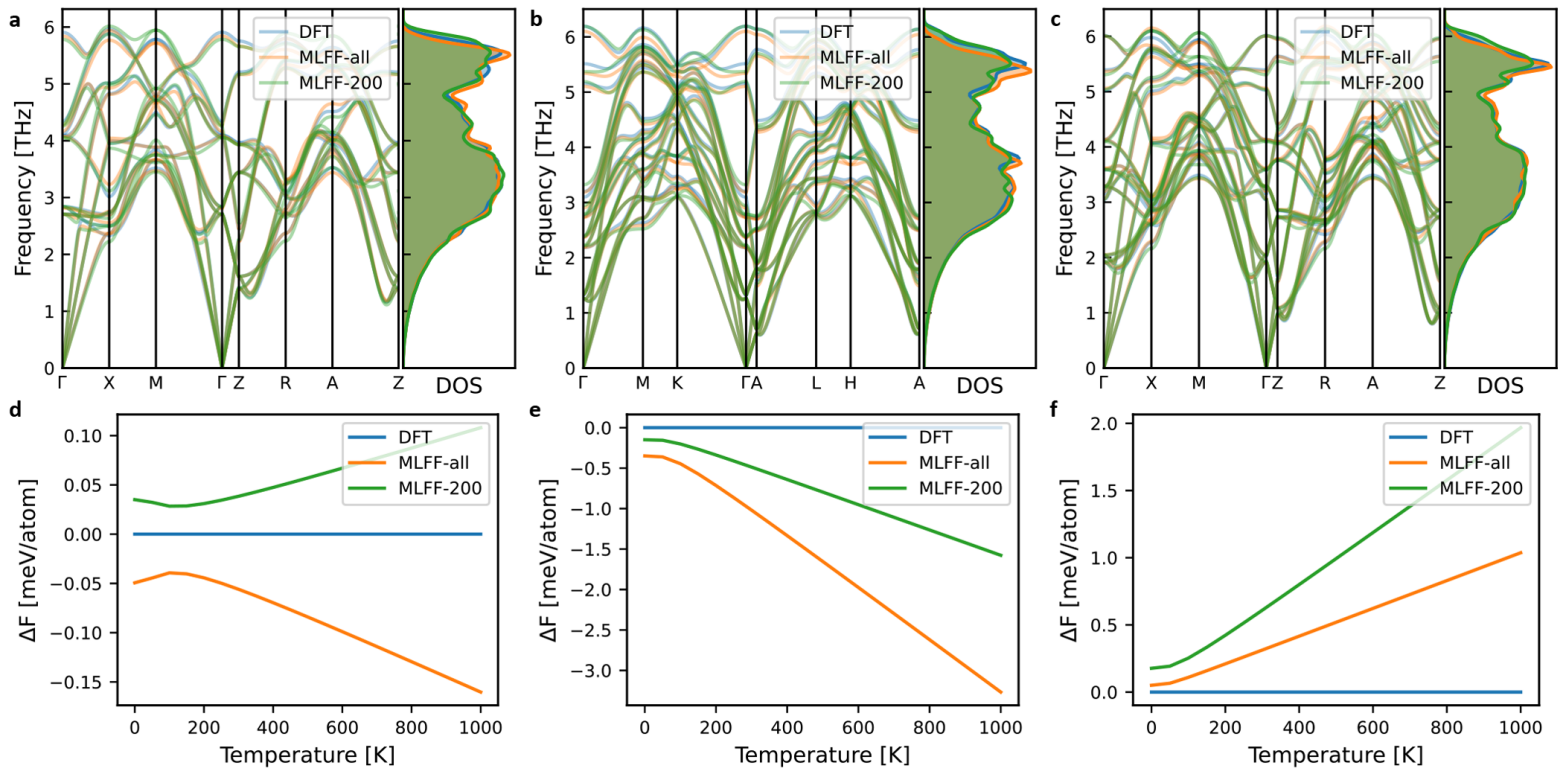
(a–c) Phonon dispersions and density of states (DOS) and (d–f) vibrational free energies of selected structures of Ag_1–*x*_Pd_*x*_ from the training set compared with those calculated from DFT. Panels d–f show the difference in vibrational free energy with respect to the DFT results (blue line). MLFF-all indicates the force field trained on all relaxation data and is the one used for production simulations, and MLFF-200 indicates the one trained on 200 relaxation trajectories. The crystal structures and compositions of these selected example cases are shown in Figure S17.

The compositional phase diagram of the Ag_1–*x*_Pd_*x*_ solid solution calculated from the three different CEs is shown in Figure 4. The main feature is a miscibility gap, but in this case, for only a narrow compositional interval toward the Pd-rich end of the phase diagram. The agreement between the calculated and experimental phase diagrams is already reasonable for the DFT-based CE. The vibrational free energy only leads to a slight stabilization of the mixed phases around *x* ≃ 0.8, but also in this case, it leads to an improved agreement with experimental results. The best agreement is again observed when end-members are constrained to DFT values, and the remaining free energies are calculated from the MLFF.

**Figure 4:**
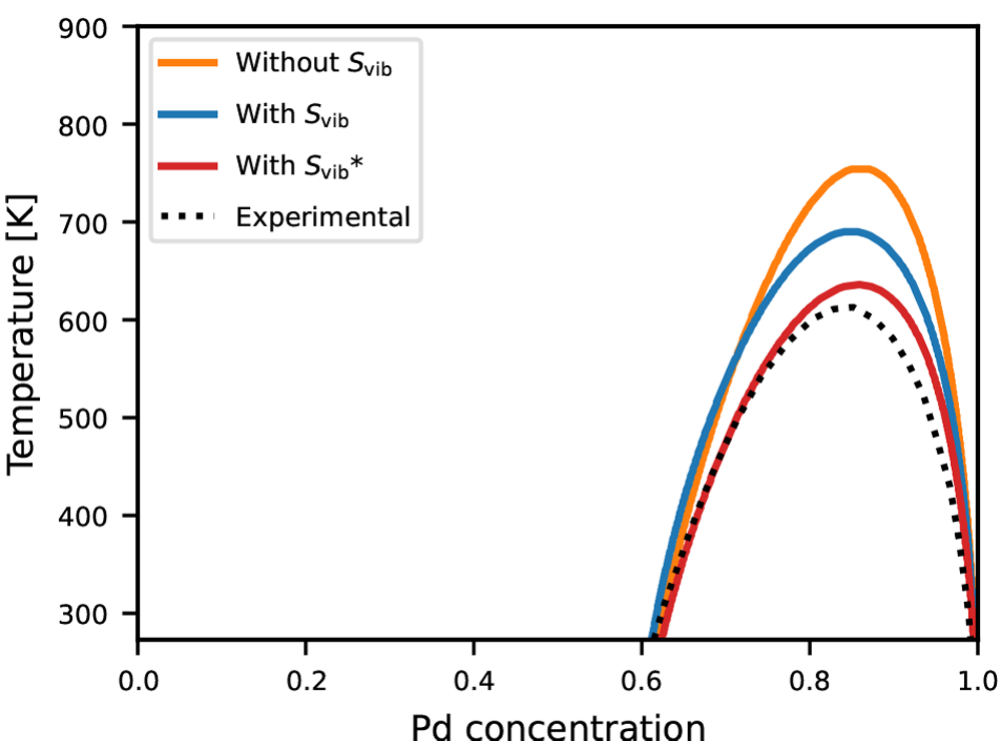
Compositional phase diagram of Ag_1–*x*_Pd_*x*_ calculated from MC simulations in the VCSGC ensemble using cluster expansions with and without inclusion of the vibrational entropy. For the ones with vibrational entropy included, the red curve marked by an asterisk is the one in which the vibrational free energies of the end-members are calculated with DFT. Experimental results from ref (35) are given for comparison.

In conclusion, we have developed a methodology for including vibrational free energies within the harmonic approximation in solid solution cluster expansions at a very low additional computational cost. This is achieved by using force fields trained on the relaxation trajectories already available for producing the data for cluster expansion in the first place. We demonstrate the viability of the methodology by calculating compositional phase diagrams for two structurally and compositionally simple (pseudo)binaries, Na_1–*x*_K_*x*_Cl and Ag_1–*x*_Pd_*x*_, as archetypical examples of ionic and metallic solid solutions, respectively. In both cases, particularly for Na_1–*x*_K_*x*_Cl, the agreement between the calculated and experimentally observed phase diagrams is significantly improved.

These observations highlight the importance of vibrational entropic contributions to the stability of alloys and solid solutions, in general. With the present methodology in hand, routine inclusion of these effects, which are typically neglected due to the computational cost, is now within reach. We note that this methodology is developed for including vibrational effects within the harmonic approximation. Anharmonic effects have been shown to contribute to the stability of complex alloys,^20^ but its inclusion in a cluster expansion would result in a large computational cost and require further benchmarking.

We envision that this methodology of training potential energy surfaces on relaxation trajectories of solid solutions will be highly relevant beyond the calculation of the vibrational free energy. For example, if representative disordered structures with thousands of atoms are constructed from Monte Carlo simulations with a CE, the trained force field can be used to relax these structures and to calculate their elastic constants, vibrational spectra, and other properties that require access to forces and stresses of structures beyond the reach of DFT. This will allow one to combine some of the benefits of on-lattice models such as the cluster expansion with the benefits of off-lattice models.^2^

In addition to other applications of such surrogate models for solid solutions, further work should focus on developing more efficient training strategies to reduce the number of configurations required. This could be inspired by the efficient methods for selecting training data for conventional cluster expansions^36−38^ and should be highly relevant for more complex compositions. For these, conventional cluster expansion modeling is already faced with challenges due to the many compositional degrees of freedom,^39−41^ and we expect this methodology to face similar challenges. Furthermore, improvements may be observed with other types of machine learning architecture such as equivariant neural network potentials.^24,42^

Finally, a similar methodology of training a force field on relaxation trajectories to calculate vibrational free energies could potentially be used when modeling defects, surfaces, and interfaces in crystals. Also for these systems, several relaxations of similar structures are performed with DFT, and vibrational free energies have been shown to be important for quantitative properties in several cases.^43,44^

## Computational Methods

*First-Principles Calculations*. Total energy calculations are performed using DFT as implemented within the Vienna ab initio simulation package (VASP). We use the projector-augmented wave method^45,46^ and the PBEsol exchange-correlation functional.^47^ A plane wave cutoff of 550 eV is used for all calculations, and forces are relaxed to 1.0 meV Å^–1^. For the Na_1–*x*_K_*x*_Cl solid solution, Gaussian smearing with a width of 0.05 eV is used, and a 6 × 6 × 6 Γ-centered **k**-point grid is used for the base cell and scaled accordingly for larger supercells. For the Ag_1–*x*_Pd_*x*_ solid solution, Methfessel–Paxton smearing with a width of 0.1 eV is used for the relaxations, while the final total energy is calculated with the tetrahedron method with Blöchl corrections. A 17 × 17 × 17 Γ-centered **k**-point grid is used for the base cell and scaled accordingly for larger supercells. For Ag_1–*x*_Pd_*x*_, spin-polarized calculations are performed initialized in the ferromagnetic state.

For each system, all symmetry unique structures with up to eight metal atoms in the unit cell are included, giving 631 structures for both systems. All relaxations are started from slightly rattled structures using the rattle function of the atomic simulation environment (ASE) with a standard deviation of 0.05 Å.^48^ Performing the relaxations without rattling results in a sampling of the potential energy surface that is too poor for training the subsequent MLFF. While the rattling does increase the number of steps in the relaxation trajectories, it also has the benefit of breaking the ideal symmetries to search for lower-energy configurations close to the ideal symmetry. The structures are resymmetrized using spglib^49^ after convergence of the first ionic relaxation, because a small deviation from ideal symmetries after the first relaxation resulted in poor numerical stability. After this, relaxation is restarted, as the plane wave basis changes with the size and shape of the simulation cell.

*Force Field Training*. The MLFFs are trained and applied using the VASP implementation,^22^ which is similar to the Gaussian approximation potential (GAP).^23^ The radial and angular descriptors are constructed using the smooth overlap of atomic positions (SOAP)^50^ with cutoffs of 5 Å for both radial and angular parts. We follow the procedure for constructing accurate force fields;^51^ first, a force field is fitted using Bayesian linear regression (BLR) with an atomic broadening of 0.3 Å and 12 radial basis functions used to expand the radial descriptor to select a diverse set of configurations from the initial data set, and second, the force field is refitted with singular-value decomposition and an atomic broadening of 0.5 Å and eight radial basis functions. The force weight is increased to 100 in the fitting procedure because we are interested in only accurate vibrational properties, whereas the internal energies for the construction of the cluster expansion are taken directly from DFT.

The training data are gathered from the first relaxation trajectory of all 631 structures, picking up every fourth step in the ionic relaxation. The relevant configurations and local environments are then selected by BLR. The MLFFs are validated in terms of out-of-sample performance by calculating the root-mean-square force error relative to DFT results from MLFFs fitted to subsets of increasing data size (up to 600 relaxation trajectories included) against the last 31 rattled structures used in the complete training set.

*Lattice Dynamics Calculations*. Lattice dynamics calculations are performed using the finite displacement method as implemented in PHONOPY.^52^ For a subset of structures for each system, the phonon dispersions are calculated with DFT for validation using the same settings as those mentioned above in supercells containing ∼100 atoms. Lattice dynamics calculations are then performed for all structures used for training the cluster expansions by using the trained MLFFs, and the vibrational free energy is calculated. 20 × 20 × 20 and 40 × 40 × 40 q-meshes are used for integrals over the phonon dispersions to calculate DOS and vibrational free energies for Na_1–*x*_K_*x*_Cl and Ag_1–*x*_Pd_*x*_, respectively. No LO–TO splitting is included in the models. Ag_1–*x*_Pd_*x*_ is metallic and thus has no contribution of LO–TO splitting. For Na_1–*x*_K_*x*_Cl, LO–TO splitting is relevant but requires access to Born effective charges and dielectric constants, which would need to be calculated for each structure, adding a large computational cost. Because the formal ionic charges of Na and K are the same, we expect the effect of LO–TO splitting to be similar on all structures, and thus, we neglect it in this study.

*Cluster Expansion Construction*. Solid solution cluster expansions (CEs) are fitted for both systems using the ICET package.^30^ For the Na_1–*x*_K_*x*_Cl solid solutions, cutoffs of 15.0 and 8.0 Å are used for two- and three-body clusters, respectively, as determined from cross validation (see Figures S1–S3). For Ag_1–*x*_Pd_*x*_, 13.0, 7.0, and 6.0 Å cutoffs are used for two-, three-, and four-body clusters, respectively, as done by Ångqvist et al.^30^ The cluster expansions are fitted with automatic relevance detection regression (ARDR) as implemented in ICET based on the scikit-learn implementation.^53^ The cluster expansion is fitted to mixing (free) energies and constrained to reproduce the (free) energies of the end-members exactly with the *get_mixing_energy_constraints* module of ICET.^30^

The static CEs are fitted against total energies of all 631 configurations, and the temperature-dependent CEs are fitted separately at each temperature by adding the vibrational free energy to each training structure. Systems with imaginary modes in their phonon dispersions are removed from the fitting, because the free energy is ill-defined in these cases.^7,54^ Phase diagrams for each system are simulated using Monte Carlo simulations in the variance-constrained semigrand canonical (VCSGC) ensemble,^29^ which allows for accurate simulations across phase boundaries, as implemented in the MCHAMMER module of ICET.^30^

## Supporting Information Available

Determination of cutoffs for cluster expansions, training and test errors for MLFFs, additional phonon dispersions from MLFF, crystal structures for the calculated phonon dispersions, comparisons of reference and cluster expansion energies, and additional phase diagrams from other models (PDF)
Transparent Peer Review report available (PDF)
