# Supplementary material for: Low-Cost Vibrational Free Energies in Solid Solutions with Machine Learning Force Fields
Source: J Phys Chem Lett. 2023 Dec 15;14(51):11618–24. doi: 10.1021/acs.jpclett.3c03083 (PMC10758113; doi:10.1021/acs.jpclett.3c03083)
Supplement: Supplementary file 1 — jz3c03083_si_001.pdf [file jz3c03083_si_001.pdf]

# Supporting Information for: Low-cost Vibrational Free Energies in Solid Solutions with Machine Learning Force Fields

Kasper Tolborg[1,2,3,*] and Aron Walsh[2,4]

[1]*Department of Chemistry and Bioscience, Aalborg University, Fredrik Bajers Vej 7H, 9220 Aalborg Ø, Denmark*
[2]*Department of Materials, Imperial College London, Exhibition Road, London SW7 2AZ, United Kingdom*
[3]*I-X, Imperial College London, Wood Lane, London W12 0BZ, United Kingdom*
[4]*Department of Physics, Ewha Womans University, Seoul 03760, Korea*
(Dated: December 6, 2023)

## SUPPORTING FIGURES

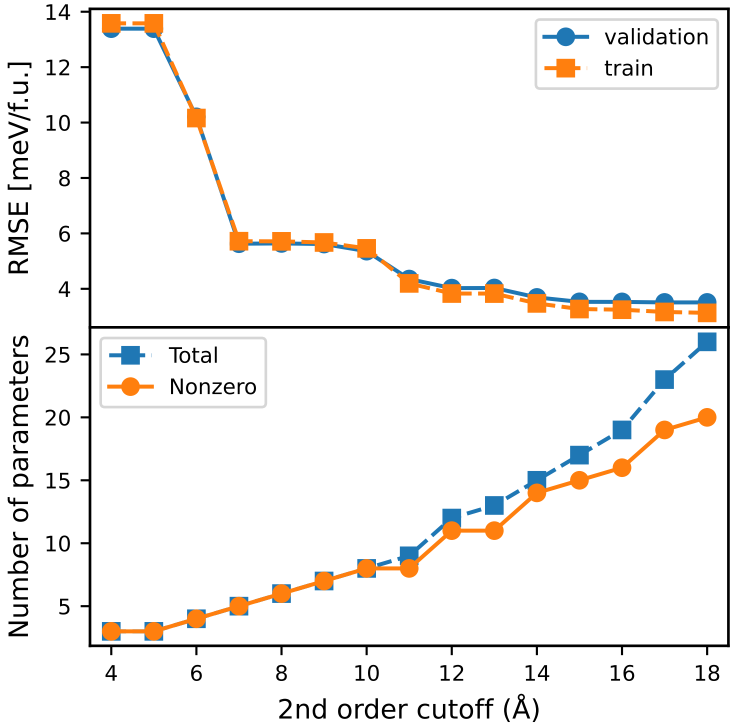


FIG. S1. Determination of 2nd order cutoff for the cluster expansion of $Na_{1-x}K_xCl$. The top panel shows the root-mean-square error (RMSE) in energy per formula unit, and the bottom panel shows the number of parameters, and the number of those that are non-zero as determined from the ARDR fitting.

* kato@bio.aau.dk

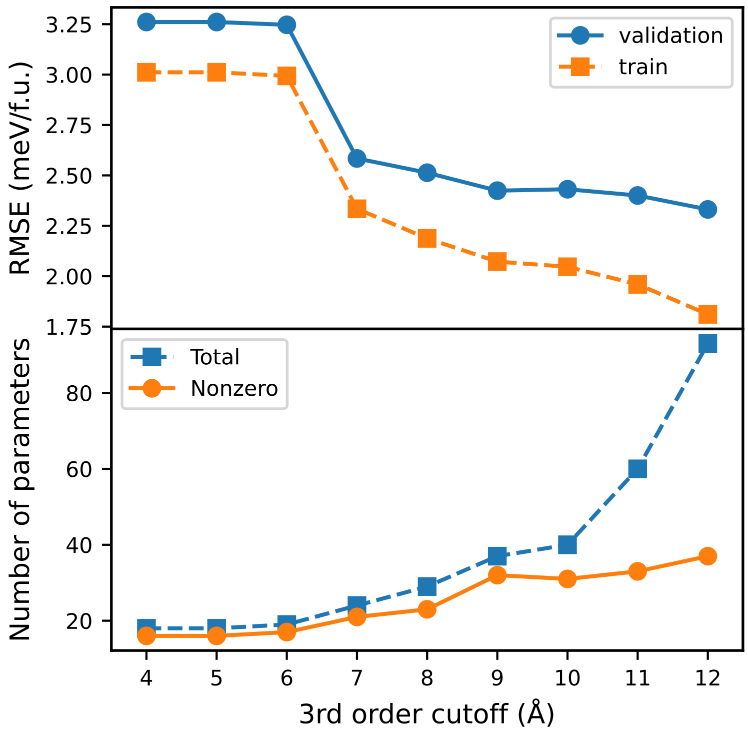


FIG. S2. Determination of 3rd order cutoff for the cluster expansion of $Na_{1-x}K_xCl$. The 2nd order cutoff is set to 15.0 Å. The top panel shows the root-mean-square error (RMSE) in energy per formula unit, and the bottom panel shows the number of parameters, and the number of those that are non-zero as determined from the ARDR fitting.

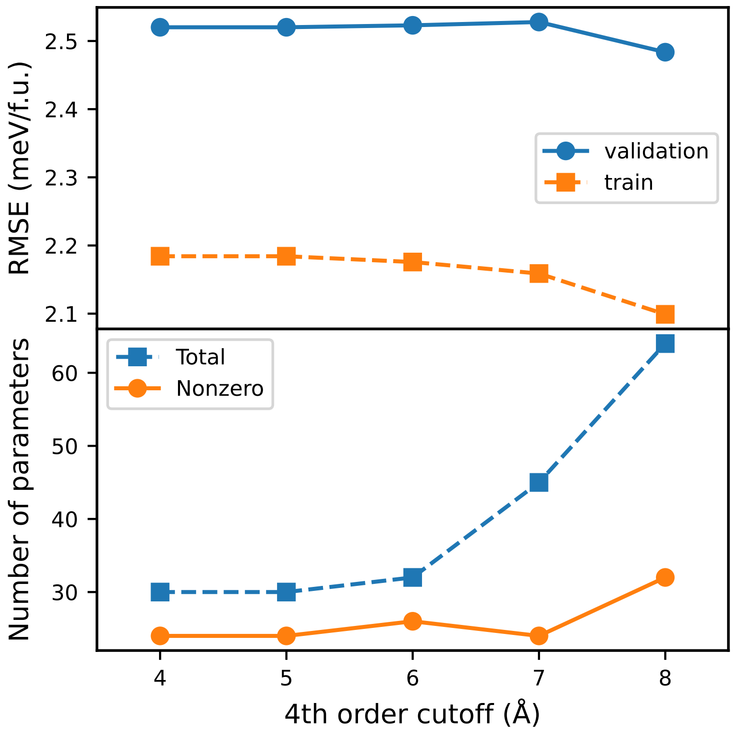


FIG. S3. Determination of 4th order cutoff for the cluster expansion of $Na_{1-x}K_xCl$. The 2nd and 3rd order cutoffs are set to 15.0 and 8.0 Å, respectively. The top panel shows the root-mean-square error (RMSE) in energy per formula unit, and the bottom panel shows the number of parameters, and the number of those that are non-zero as determined from the ARDR fitting.

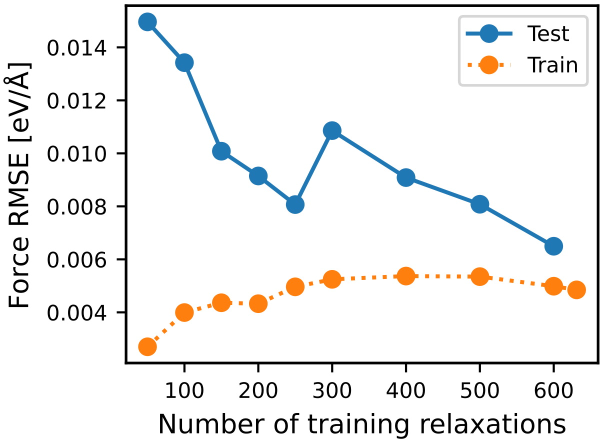


FIG. S4. Force errors of training and test sets for the MLFF of $Na_{1-x}K_xCl$ for different sizes of training sets. The training error is the internal force error on the full training set, and the test error is the force error on single-point calculations of rattled structures of the last 31 mixed structures. Thus, test errors are only meaningful up to a training set of 600 relxation trajectories.

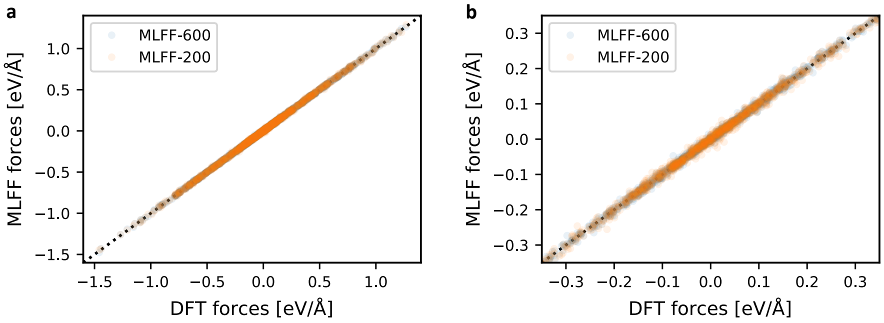


FIG. S5. Parity plot of the forces between MLFF and DFT for $Na_{1-x}K_xCl$ on the test set. The test forces are the ones of single-point calculations of rattled structures of the last 31 mixed structures. Thus, the comparison is only meaningful up to a training set of 600 relaxation trajectories.

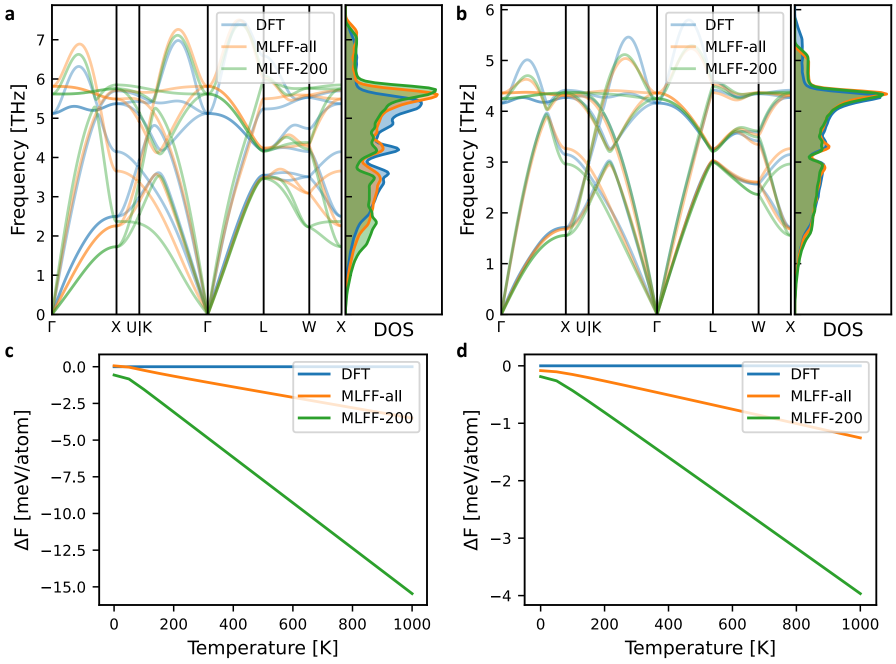


FIG. S6. Phonon dispersions, density of states and vibrational free energies of **a** and **c** NaCl and **b** and **d** KCl.

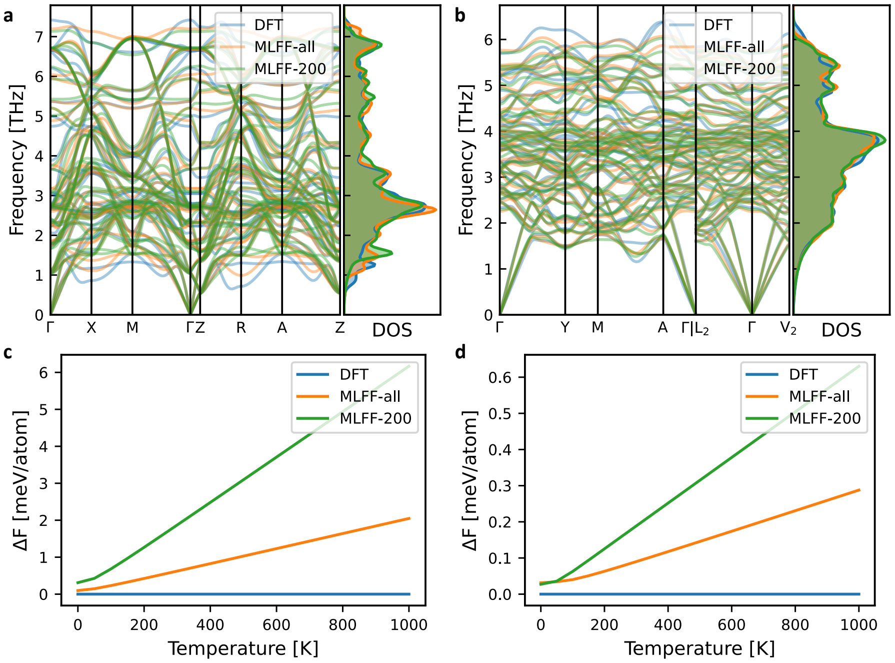


FIG. S7. Phonon dispersions, density of states and vibrational free energies of two additional mixed $Na_{1-x}K_xCl$ structures from the training set.

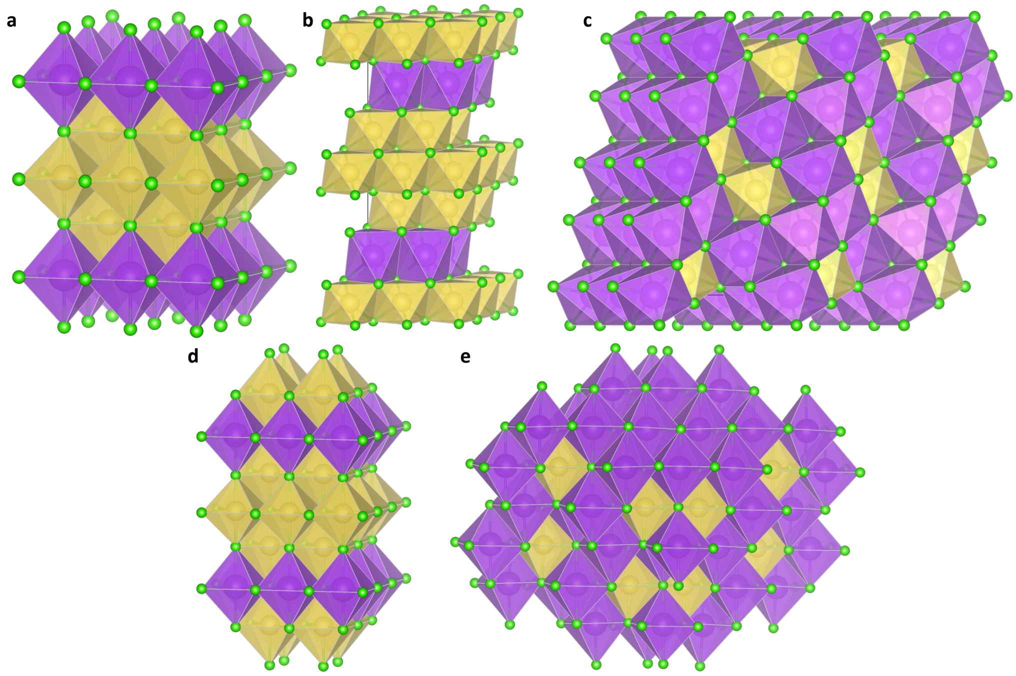


FIG. S8. Crystal structures of the example cases for validation of the phonon dispersions and vibrational free energies in the $Na_{1-x}K_xCl$ system. Na is shown in yellow, K in purple, and Cl in green. **a**, **b** and **c** shows the structures corresponding to the phonon dispersions in Fig. 1**a**, **b** and **c** in the main text, and **d** and **e** shows the ones from Fig. S7**a** and **b**. The compositions are $Na_3KCl_4$, $Na_4K_2Cl_6$, $Na_2K_4Cl_6$, $Na_4K_2Cl_6$, and $Na_2K_4Cl_6$, respectively.

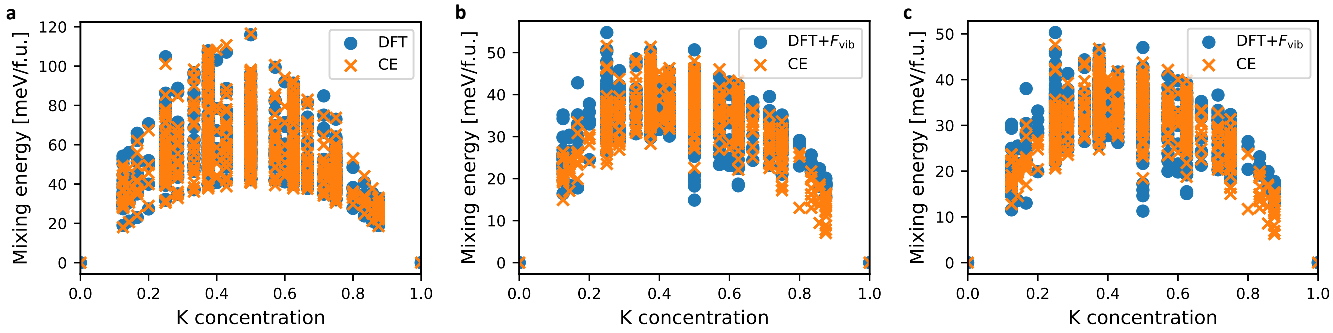


FIG. S9. Comparison of reference energies from DFT ($+F_{\text{vib}}$) and the fitted cluster expansion for $Na_{1-x}K_xCl$. **a** Internal energies from DFT, **b** Including vibrational free energy from MLFF at 800 K and **c** Including vibrational free energy from MLFF and from DFT for end-members at 800 K. Root-mean square errors from 10-fold cross-validation are 2.5, 3.7 and 4.1 meV per formula unit (f.u.). These cluster expansion models are used in the main text to produce the phase diagram in Fig. 2.

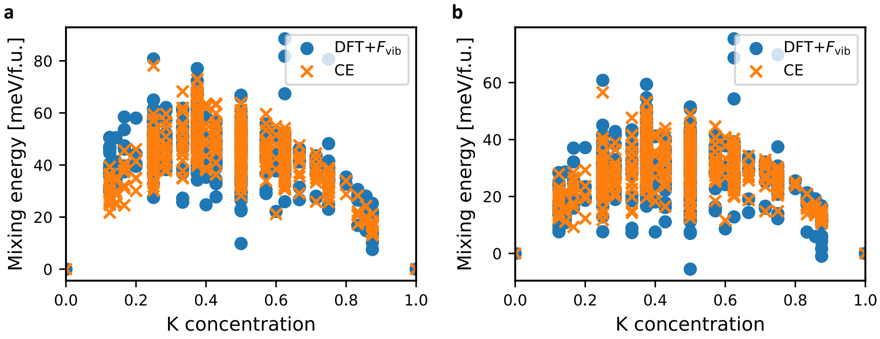


FIG. S10. Comparison of reference energies from DFT+$F_{\text{vib}}$ and the fitted cluster expansion for $Na_{1-x}K_xCl$ using the the MLFF-200 force field, which is only trained on the first 200 relaxation trajectories. **a** Including vibrational free energy from MLFF at 800 K and **b** Including vibrational free energy from MLFF and from DFT for end-members at 800 K. These cluster expansion models are used to produce the phase diagram in Fig. S12

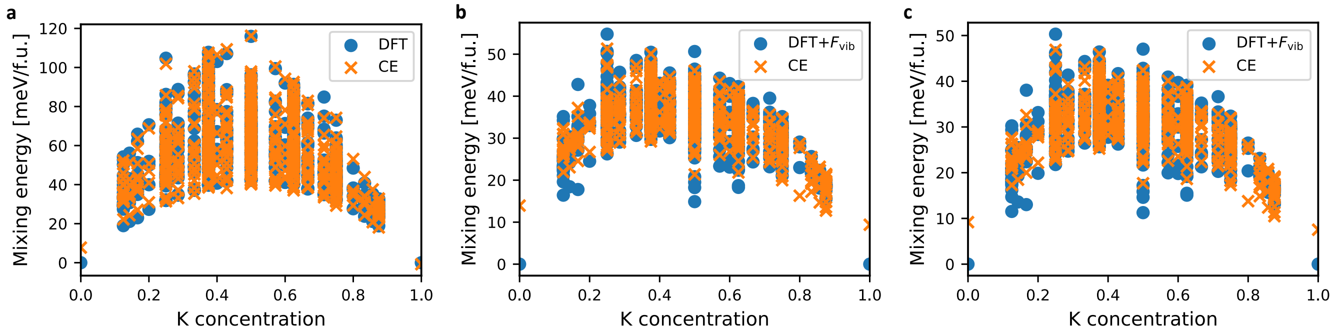


FIG. S11. Comparison of reference energies from DFT ($+F_{\text{vib}}$) and the fitted cluster expansion for $Na_{1-x}K_xCl$, where the cluster expansion is not constrained to reproduce the (free) energy of the end-members exactly. **a** Internal energies from DFT, **b** Including vibrational free energy from MLFF at 800 K and **c** Including vibrational free energy from MLFF and from DFT for end-members at 800 K. These cluster expansion models are used to produce the phase diagram in Fig. S13

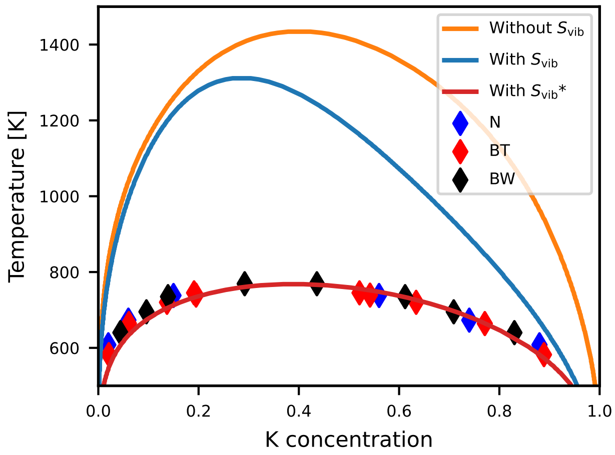


FIG. S12. Compositional phase diagram of $Na_{1-x}K_xCl$ calculated from MC simulations in the VCSGC ensemble using cluster expansions with and without inclusion of vibrational entropy, where the vibrational entropy is based on the MLFF-200 force field, which is only trained on the first 200 relaxation trajectories. For the ones with vibrational entropy included, the red curve marked by an asterisk (*) is the one where the vibrational free energies of the end-members are calculated with DFT. Note that the phase diagram using only MLFF free energies is of poor quality, especially in the Na-rich region. However, as seen from Fig. S6c, this MLFF-200 gives poor results compared to DFT for pure NaCl. Using the DFT free energy for pure NaCl gives much better agreement with experiment. Experimental results are given for comparison as in Fig. 2 in the main text.

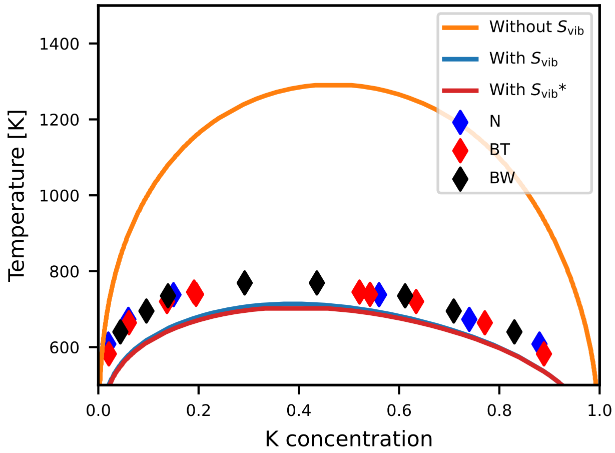


FIG. S13. Compositional phase diagram of $Na_{1-x}K_xCl$ calculated from MC simulations in the VCSGC ensemble using cluster expansions with and without inclusion of vibrational entropy, where the cluster expansions are not constrained to reproduce the (free) energies of the end-members exactly. For the ones with vibrational entropy included, the red curve marked by an asterisk (*) is the one where the vibrational free energies of the end-members are calculated with DFT. Experimental results are given for comparison as in Fig. 2 in the main text.

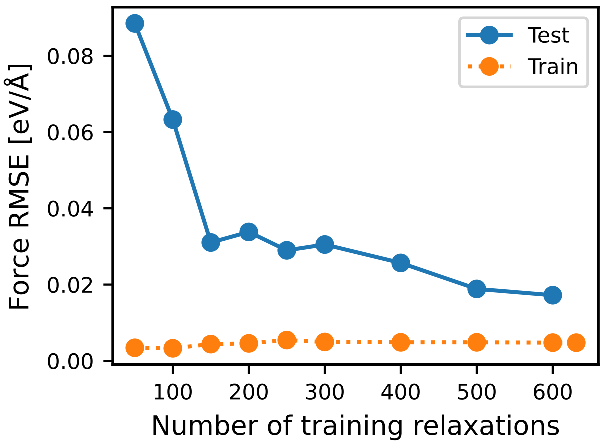


FIG. S14. Force errors of training and test sets for the MLFF of $Ag_{1-x}Pd_x$ for different sizes of training sets. The training error is the internal force error on the full training set, and the test error is the force error on single-point calculations of rattled structures of the last 31 mixed structures. Thus, test errors are only meaningful up to a training set of 600 relxation trajectories.

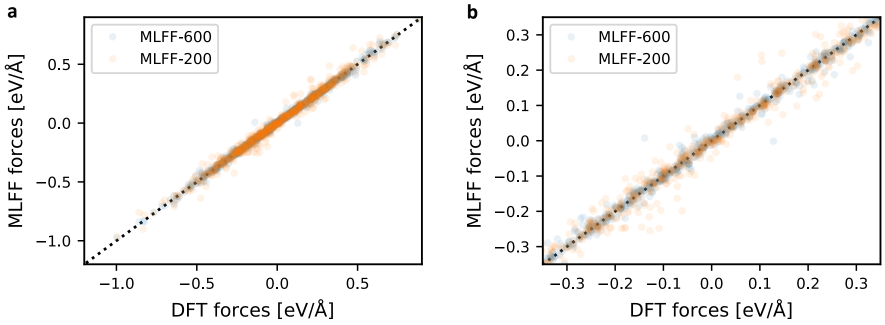


FIG. S15. Parity plot of the forces between MLFF and DFT for $Ag_{1-x}Pd_x$ on the test set. The test forces are the ones of single-point calculations of rattled structures of the last 31 mixed structures. Thus, the comparison is only meaningful up to a training set of 600 relaxation trajectories.

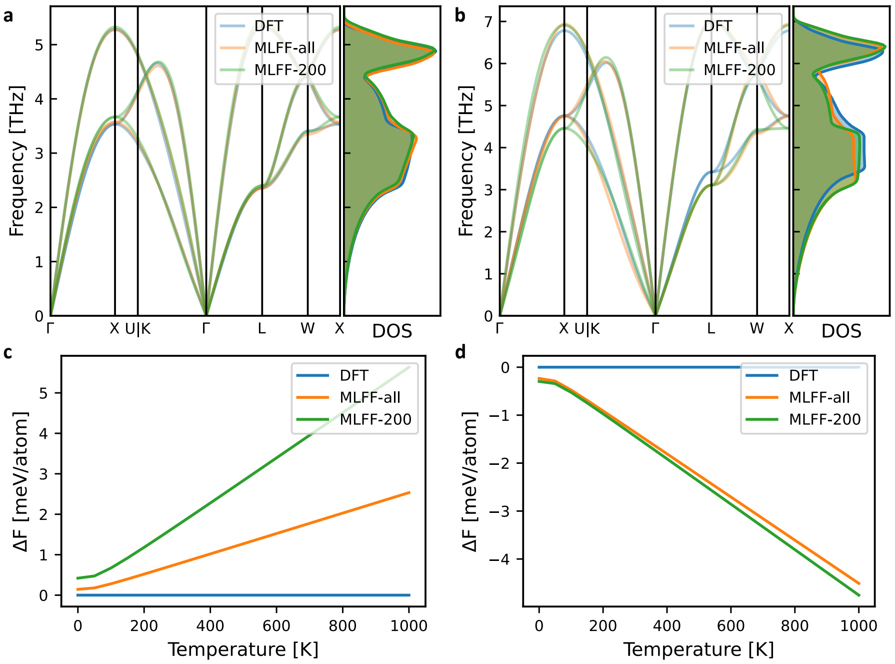


FIG. S16. Phonon dispersions, density of states and vibrational free energies of **a** and **c** Ag and **b** and **d** Pd.

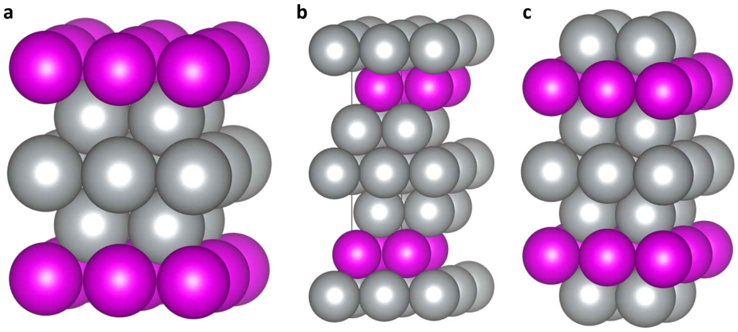


FIG. S17. Crystal structures of the example cases for validation of the phonon dispersions and vibrational free energies in the $Ag_{1-x}Pd_x$ system. Ag is shown in grey, and Pd is shown in pink. **a**, **b** and **c** shows the structures corresponding to the phonon dispersions in Fig. 1**a**, **b** and **c** in the main text. The compositions are $Ag_3Pd$, $Ag_4Pd_2$ and $Ag_4Pd_2$ respectively.

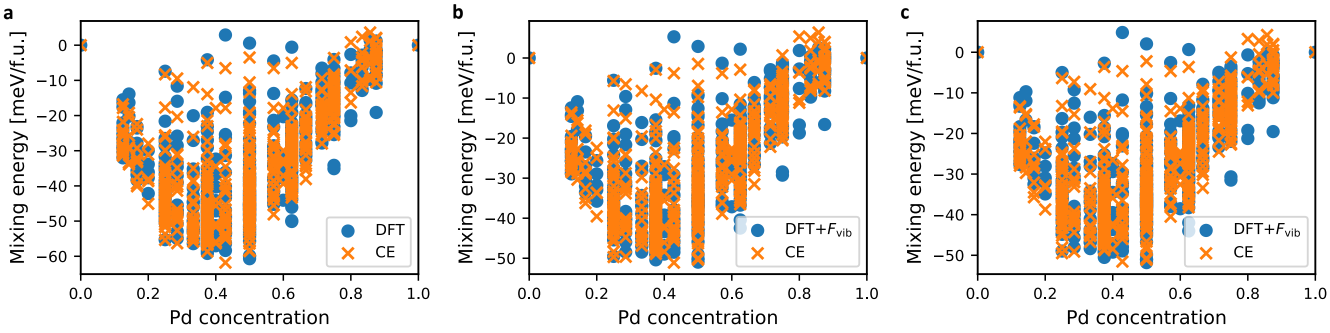


FIG. S18. Comparison of reference energies from DFT ($+F_{vib}$) and the fitted cluster expansion for $Ag_{1-x}Pd_x$. **a** Internal energies from DFT, **b** Including vibrational free energy from MLFF at 800 K and **c** Including vibrational free energy from MLFF and from DFT for end-members at 800 K. Root-mean square errors from 10-fold cross-validation are 3.1, 3.0 and 3.0 meV per formula unit (f.u.)
