# Supplementary material for: Low-Cost Vibrational Free Energies in Solid Solutions with Machine Learning Force Fields
Source: J Phys Chem Lett. 2023 Dec 15;14(51):11618–24. doi: 10.1021/acs.jpclett.3c03083 (PMC10758113; doi:10.1021/acs.jpclett.3c03083)
Supplement: Supplementary file 2 — jz3c03083_si_002.pdf [file jz3c03083_si_002.pdf]

jz-2023-03083s.R1

Name: Peer Review Information for "Low-Cost Vibrational Free Energies in Solid Solutions with Machine Learning Force Fields"

First Round of Reviewer Comments

Reviewer: 1

Comments to the Author

The key advancement presented in this work is the inclusion of vibrational energy within the cluster expansion methodology for studying alloys and solid solutions. This ties nicely into the recent trend where modern machine learning and regression advancements have added new interest to cluster expansions, and the authors' work is a very valuable addition to this narrative.

This study has the potential for delivering significant improvements in the precision of models for alloys and solid solutions for a small computational overhead relative to previous methods, which is undoubtedly of great relevance to the community of physical and materials chemistry.

I propose a few points for the authors to consider before resubmission, which I believe do not constitute major barriers to publication.

Firstly, in the first paragraph it is implied that this approach could allow us a better understanding of high-entropy and many-component systems. Yet, these systems pose inherent challenges within "standard" cluster expansion due to their compositional and configurational complexity [1, 2, 3]. It appears that the surrogate model applied may face similar or more significant scalability issues, and I think this casual association is slightly misleading. I would prefer the link not implied, or that it was qualified.

Regarding the AgPd phase diagram, the inclusion of vibrational energy introduces an interesting qualitative discrepancy when compared to experimental findings, with the emergence of a plateau absent in empirical data. As this is a proof-of-concept work, I don't see a need for immediate resolution in this manuscript. However, an acknowledgment of this discrepancy, discussing potential contributors such as model selection or training data, would be beneficial for the reader

Lastly, for enhancing the manuscript, I suggest including a reference to iterative structure selection based on predicted uncertainties, which could further optimise the training data generation and efficacy of the modelling process [4].

Finally, I'd just like to reiterate the fact I think this is an exciting development in the treatment of solid solutions for the materials chemistry community, and I am excited to see how the authors and the community go on to build on these developments.

[1] https://doi.org/10.1016/j.pmatsci.2022.101018

[2] https://doi.org/10.1038/s41524-022-00818-3

[3] https://doi.org/10.1039/D3TA02088F

[4] https://doi.org/10.1088/2515-7655/abf9ef.

Reviewer: 2

Comments to the Author

Review attached

Review

1. What is the major advance reported in the paper?

The paper ”Low-Cost Vibrational Free Energies in Solid Solutions with Machine Learning Force Fields” concerns a new computational approach for modeling vibrational free energies in alloys and mixed systems. The inclusion of vibrational free energies when modeling alloys can be very important for many systems, as have previously been shown and is shown by the authors. Therefore, the development and improving methods to carry out such modeling more efficiently is important and relevant for computational materials science in general.

2. What is the immediate significance of this advance?

The methodology outline in the manuscript will allow researchers to more accurately model alloys and mixed systems by taking into account vibrational free energy in a computational efficient way. The convergence testing and detailed analysis of the accuracy of the models will be helpful for future studies on this topic.

3. Technical suggestions

I recommend the paper to be published after major revision regarding the following points.

1. I wonder if the authors could discuss the possible extension of this approach beyond mixing energies in mixed systems, for example could one employ a this approach to defect formation energies, surface interface energies etc? In these cases one typically also relaxes many structures to obtain their minimum energy in order to e.g. calculate defect formation energies. Do the authors think it would be possible to train MLFFs on these relaxation trajectories and ”for free” get the defect vibrational formation free energy, or would this not be feasible?

2. In total quite a number of training structures is used for training the final MLFF. Do the authors have some arguments why using the relaxation trajectories is better than e.g. spending the same amount of computational resources to train a MLFF using a conventional approach?

3. I think would be useful and instructive for readers if the authors could include figures of the total and vibrational mixing free energy as a function of concentration for some select temperatures. This would give some context on how small changes in the vibrational free energy would for example shift a phase-boundary by say 100K. Additionally, this would help understand how small (or large) the vibrational free energy errors are shown in Fig 1d,e,f) and Fig 3d,e,f). Furthermore, could the authors calculate the phase diagram obtained from the MLFF-200 model? Again, I think this would help the reader understand how small energy and entropy errors propagate into complex thermodynamic properties such the phase diagram.

4. The harmonic approximation can lead to significant errors for strongly anharmonic materials and/or at high temperatures. Therefore it would be interesting to see, for maybe just for one or a few configurations, how accurate the harmonic approximation is at the relevant temperatures compared to the full anharmonic free energy for the system studied. This would help further validate the approach in the manuscript.

5. The authors report ”Generally, we observe that the mixed phases are significantly stabilised due to vibrational free energy.”, is there some explanation for this? The mixing vibrational free energy of a configuration can often be anti-correlated with its mixing energy, i.e. strong stiff bonds yields a low energy but also high frequencies and thus a low vibrational entropy (as observed in previous work where cluster expansion have been combined with vibrational free energies). Is this something the authors could explore further to provide an explanation for their observation that the inclusion of vibrational effects stabilizes mixed phases?

6. I did not understand the authors discussion on the error cancellation in the pure phases, can the authors expand and clarify this? For cluster expansions in general the error may be larger for the end members due to the large bias in the training structures towards concentrations around 50% and almost no data points close to x=0 or x=1.

7. What is the motivation for using the constraints when constructing the CEs? And what are the consequences (in terms of mixing energies and thermodynamic properties) of applying such

constraints? For example maybe showing the phase diagram with and without the constraints would help motivate the usage of constraints. Additionally, I think the authors should specify how these constraints are imposed as there are many different ways one can do this.

8. Can the authors spell out more precisely what structure the phonon dispersions are calculated for in Fig 1a,b,c) and Fig3a,b,c), e.g. what are he concentrations, are the structures completely random or ordered etc?

9. Showing the parity plots of e.g. energy and forces for the MLFF would be good for the reader. It is for example hard to evaluate if an error of 4.8meV/Å is small or not, specially since most of the training structures likely have forces close to zero?

10. It would be helpful with some additional details regarding the relaxation process. Why are the structures rattled and then relaxed, and why then symmetrized and then relaxed again? And how is the symmetrization done?

11. It would be useful some more information regarding the phonopy calculations, e.g. how large was the q-point mesh used? Do the authors not include LO-TO splitting in the calculations (is this not relevant for the free energy)?

12. The term ”end-members” was introduced without any explanation.

13. A suggestion is to also cite sklearn and possibly sources therein for the implementation of ARDR.

Author's Response to Peer Review Comments:

Dear Prof. Editor,

We thank you for considering our manuscript for publication in the Journal of Physical Chemistry Letters. Below, we will give a point-by-point response to the reviewers' comments. The original reviewer comments are marked in blue, our responses in black, and changes to the manuscript highlighted in yellow.

We have also submitted two versions of the manuscript, one with changes highlighted in yellow, and one without highlighting.

In addition, we have taken into account the style changes requested by the editor.

Yours Sincerely,
Kasper Tolborg and Aron Walsh

Reviewer 1

The key advancement presented in this work is the inclusion of vibrational energy within the cluster expansion methodology for studying alloys and solid solutions. This ties nicely into the recent trend where modern machine learning and regression advancements have added new interest to cluster expansions, and the authors' work is a very valuable addition to this narrative.

This study has the potential for delivering significant improvements in the precision of models for alloys and solid solutions for a small computational overhead relative to previous methods, which is undoubtedly of great relevance to the community of physical and materials chemistry.

I propose a few points for the authors to consider before resubmission, which I believe do not constitute major barriers to publication.

We thank the reviewer for their positive comments, and we will respond to their points below.

Firstly, in the first paragraph it is implied that this approach could allow us a better understanding of high-entropy and many-component systems. Yet, these systems pose inherent challenges within "standard" cluster expansion due to their compositional and configurational complexity [1, 2, 3]. It appears that the surrogate model applied may face similar or more significant scalability issues, and I think this casual association is slightly misleading. I would prefer the link not implied, or that it was qualified.

We thank the reviewer for pointing this out. We did not intend to imply that our method would be easily applicable to high-entropy compounds for which the standard cluster expansion is struggling. We have slightly reworded the first paragraph, so we refer to high entropy systems only as a motivation for studying alloys and solid solutions, whereas the first principles calculation of phase diagrams now refers to alloys and solid solutions to not imply that our method is targeted high entropy systems. Furthermore,

we have expanded upon our discussion in the final paragraph on the future work and applicability to more complex systems stating now (where the three references are the ones suggested by the reviewer): "For these, conventional cluster expansion modelling is already faced with challenges due to the many compositional degrees of freedom [1,2,3], and we expect the present methodology to face similar challenges."

Regarding the AgPd phase diagram, the inclusion of vibrational energy introduces an interesting qualitative discrepancy when compared to experimental findings, with the emergence of a plateau absent in empirical data. As this is a proof-of-concept work, I don't see a need for immediate resolution in this manuscript. However, an acknowledgment of this discrepancy, discussing potential contributors such as model selection or training data, would be beneficial for the reader

We thank the referee for pointing this out and we are happy to address it. The plateau was merely a result of a too coarse grid on the temperature axis for the interpolation used when identifying the phase boundaries from the data arising from the Monte Carlo simulations. We have updated the figures both for AgPd and (Na/K)Cl with a finer temperature spacing, which leads to a disappearance of the plateau.

Lastly, for enhancing the manuscript, I suggest including a reference to iterative structure selection based on predicted uncertainties, which could further optimise the training data generation and efficacy of the modelling process [4].

We thank the reviewer for suggesting this reference. We have added it in the final section as another example (besides Ref. 36 and 37 in the original manuscript) of an efficient method for selecting training data for the cluster expansion.

Finally, I'd just like to reiterate the fact I think this is an exciting development in the treatment of solid solutions for the materials chemistry community, and I am excited to see how the authors and the community go on to build on these developments.

We again thank the referee for this positive and encouraging comment, and we share the excitement for future developments in the field.

[1] https://doi.org/10.1016/j.pmatsci.2022.101018
[2] https://doi.org/10.1038/s41524-022-00818-3 [3] https://doi.org/10.1039/D3TA02088F [4] https://doi.org/10.1088/2515-7655/abf9ef.

Reviewer 2

1. What is the major advance reported in the paper?
The paper "Low-Cost Vibrational Free Energies in Solid Solutions with Machine Learning Force Fields" concerns a new computational approach for modeling vibrational free energies in alloys and mixed systems. The inclusion of vibrational free energies when modeling alloys can be very important for many systems, as have previously been shown and is shown by the authors. Therefore, the development and improving methods to carry out such modeling more efficiently is important and relevant for computational materials science in general.

2. What is the immediate significance of this advance?
The methodology outline in the manuscript will allow researchers to more accurately model alloys and mixed systems by taking into account vibrational free energy in a computational efficient way. The convergence testing and detailed analysis of the accuracy of the models will be helpful for future studies on this topic.

We thank the reviewer for their positive comments and technical suggestions. We will address those point by point below.

3. Technical suggestions
I recommend the paper to be published after major revision regarding the following points.

1. I wonder if the authors could discuss the possible extension of this approach beyond mixing energies in mixed systems, for example could one employ a this approach to defect formation energies, surface interface energies etc? In these cases one typically also relaxes many structures to obtain their minimum energy in order to e.g. calculate defect formation energies. Do the authors think it would be possible to train MLFFs on these relaxation trajectories and ”for free” get the defect vibrational formation free energy, or would this not be feasible?

This is a very good suggestion, and we indeed hope that a similar methodology would be feasible for defects, surfaces and interfaces for which the computational workflow with many relaxations of similar structures is quite similar to the workflow here. We have added a paragraph at the end of the manuscript to highlight these potential applications, although we note that they will require extensive testing before widely employed:

“Finally, a similar methodology of training a force field on relaxation trajectories to calculate vibrational free energies could potentially be used when modelling defects, surfaces and interfaces in crystals. Also for these systems, several relaxations of similar structures are performed with DFT, and vibrational free energies have been shown to be important for quantitative properties in several cases (Mosquera-Lois et al., 2023; Kempisty et al., 2019)”

2. In total quite a number of training structures is used for training the final MLFF. Do the authors have some arguments why using the relaxation trajectories is better than e.g. spending the same amount of computational resources to train a MLFF using a conventional approach?

We agree that quite a lot of training structures are used for the final MLFF, and that often fewer training structures are used for constructing cluster expansions of (pseudo)binary systems. However, we note that all relaxations are performed on small supercells, meaning that all DFT calculations are relatively fast. Using a more conventional approach for MLFFs of training on molecular dynamics trajectories typically includes simulations in larger

supercells, suffering from the poor size scaling of DFT. Furthermore, using the enumerated symmetry-unique small supercells ensures that all relevant local environments are properly sampled. As we note in the final paragraphs, future studies should indeed investigate if better selection of training data – both for the cluster expansion and trajectories for the MLFF – is possible.

As noted in the response to comment 3, we have now also included a phase diagram calculated from the MLFF-200 force field, which also results in good agreement with experimental observations. Thus, it is not unlikely that smaller data sets can be used in future with this methodology.

3. I think would be useful and instructive for readers if the authors could include figures of the total and vibrational mixing free energy as a function of concentration for some select temperatures. This would give some context on how small changes in the vibrational free energy would for example shift a phase-boundary by say 100K. Additionally, this would help understand how small (or large) the vibrational free energy errors are shown in Fig 1d,e,f) and Fig 3d,e,f). Furthermore, could the authors calculate the phase diagram obtained from the MLFF-200 model? Again, I think this would help the reader understand how small energy and entropy errors propagate into complex thermodynamic properties such the phase diagram.

The mixing (internal) energies as a function of concentration are given in Fig. S9a and S18a (figure numbers in the revised manuscript), and Fig. S9b+c and S18b+c gives the mixing energy including vibrational contributions at 800 K for both systems. From these, it can, for example, be seen that the difference between Fig. S18a and S18c (~10 meV/f.u.) is enough to shift the miscibility gap by ~150 K in AgPd.

To test the effects of the accuracy of the force field and vibrational free energy, we have calculated the phase diagram with vibrational contributions using the MLFF-200 force field for the (Na/K)Cl system. The cluster expansion reconstruction and the calculated phase diagram are shown in Fig. S10 and S12. We note that MLFF-200 gives a poor fit to pure NaCl (Fig. S6), and thus the phase diagram using only MLFF free energies is very poor. However, using the DFT vibrational free energies for the end-members again gives good agreement with experiment.

Furthermore, we have calculated the phase diagram using a cluster expansion, which is not constrained to reproduce the (free) energy of the end-members exactly as suggested in comment 7. The results are shown in Fig. S11 and S13 and furthermore highlights how differences in the cluster expansion fitting can lead to differences in the resulting phase diagram.

We have added the following paragraph to discuss these effects:

"To investigate the effect of accuracy of the force field and of constraining the energy of the end-members, we provide two additional test: (i) we use the force field only trained on the first 200 relaxation trajectories (denoted MLFF-200 in Fig. 1); and (ii) we follow the same procedure as above, but do not enforce the cluster expansion to reproduce the (free) energies of the pure phases exactly. The cluster expansion fit and calculated phase diagram of (i) are shown in Fig. S10 and S12. We note that the vibrational free energy of NaCl is poorly reproduced with MLFF-200 (Fig. S6), and thus only relying on the MLFF gives a poor phase diagram. However, using the vibrational free energy from DFT for the end-members results in a phase diagram in good agreement with experiment also for this force field, which is trained on significantly less data. Similarly, the cluster expansion fit and calculated phase diagram of (ii) are shown in Fig. S11 and S13. We note that this leads to large deviations in the (free) energy of the end-members between cluster expansion and reference energies, since the large amount of data for mixed phases bias the cluster expansion towards these. Generally, this leads to smaller mixing (free) energies (Fig. S11) and results in a miscibility gap at lower temperatures (Fig. S13) compared to the constrained model shown in Fig. 2.

These different tests highlight the precision of the phase diagram reconstruction resulting from the different model choices, both in terms of conventional cluster expansion choices such as whether to constrain the model to end-members, and in terms of accuracy of the vibrational free energy. In all cases, inclusion of vibrational entropy significantly improves the agreement with experimental results."

4. The harmonic approximation can lead to significant errors for strongly anharmonic materials and/or at high temperatures. Therefore it would be interesting to see, for maybe just for one or a few configurations, how accurate the harmonic approximation is at the relevant temperatures compared to the full anharmonic free energy for the system studied. This would help further validate the approach in the manuscript.

We agree with the reviewer that anharmonic effects are often important at high temperatures. However, we consider a treatment of anharmonic effects beyond the scope of the current proof-of-concept paper for the following reasons:

(i) Comparison of anharmonic and harmonic absolute free energies are not possible as the compared phases are then not treated on an equal footing. Thus, many configurations would need to be calculated at the same anharmonic level of theory to gain insight into whether anharmonicity would have an effect on mixing energies, or if the effects are similar for all systems.

(ii) Inclusion of anharmonic effects comes with a significant computational overhead, even if the calculation of force constants in relatively fast, especially when 100s or 1000s of structures need to be evaluated. This means that our methodology is only feasible within the harmonic approximation for which the computational overhead of the phonon calculations (after the force constants are determined) is relatively light.

(iii) Calculation of anharmonic effects would require benchmarking of the accuracy of higher order force constants (or derived effective harmonic phonon dispersions and free energies) against DFT to understand if the calculated free energies are meaningful. This would add a significant computational cost to the present study as well as other studies using the method.

In total, investigating anharmonic effects is beyond the present scope, but we acknowledge that they may have an effect. We have thus added a paragraph towards the end of the paper describing that the present methodology is only feasible within the harmonic approximation, which we, however, believe is already a significant step forward compared to neglecting vibrational contributions completely. The paragraph reads:

"We note that the present methodology is developed for including vibrational effects within the harmonic approximation. Anharmonic effects have been shown to contribute to the stability of complex alloys (Grabowski et al., 2019), but its inclusion in a cluster expansion would significantly increase the computational cost and require further benchmarking."

5. The authors report "Generally, we observe that the mixed phases are significantly stabilised due to vibrational free energy.", is there some explanation for this? The mixing vibrational free energy of a configuration can often be anti-correlated with its mixing energy, i.e. strong stiff bonds yields a low energy but also high frequencies and thus a low vibrational entropy (as observed in previous work where cluster expansion have been combined with vibrational free energies). Is this something the

authors could explore further to provide an explanation for their observation that the inclusion of vibrational effects stabilizes mixed phases?

The comment cited by reviewer refers to he (Na/K)Cl system for which vibrational contributions stabilize the mixed phases. During the revision, we realized that we had also mentioned that the inclusion of vibrational free energy stabilizes the mixed phases of AgPd. As one can see from Fig. S10, the mixed phases at around x=0.5 are in fact slightly destabilized at 800 K, which is the reason why the mixed phases around x=0.8 experience a relative stabilization by vibrational entropy and are therefore predicted to be miscible at lower temperatures. We have corrected this observation in the main text, so it now reads:

"Compared to the ´$Na_{1-x}K_xCl$ case, a smaller effect of vibrational entropy and a different kind of stabilisation is observed. In $Ag_{1-x}Pd_x$, the structures around x~0.5 are slightly destabilised relative to the end-members at elevated temperatures, which results in a relative stabilisation of mixed phases around x~0.8 as will be clear from the phase diagram."

Regarding the idea that mixed phases are often stabilized by vibrational entropy, the reviewer is completely right that there is often a tendency that higher energy configuration have larger vibrational entropy, since weaker bonds is the origin of both. However, as shown by Manzoor et al. (2018) (Ref. 10 in both original and revised manuscript), there are also several examples in which the opposite is true. The present study did not intend to deeply investigate this relation and its origin, and we do not believe that studying the relations deeply in the two systems investigated here would add significantly to our general understanding of this phenomenon, since several counterexamples of effects in the opposite direction exist. We agree that the subject merits further study in a wider range of systems.

6. I did not understand the authors discussion on the error cancellation in the pure phases, can the authors expand and clarify this? For cluster expansions in general the error may be larger for the end members due to the large bias in the training structures towards concentrations around 50% and almost no data points close to x=0 or x=1.

The lower degree of error cancellation in the pure phases as mentioned in the last paragraph on page 2 refers to their vibrational free energies, and not the cluster expansion. We simply mean that when relatively few phonon branches are present, the vibrational free energy, which is calculated as an integral over these, becomes much more prone to small deviations in their frequencies. For systems with many phonon branches, one can expect some cancellation of errors in the vibrational free energy, if some phonon frequencies are predicted a bit too high and others a bit too low, which will often be the case. We have edited the paragraph to clarify this, so it now reads:

"The error in the vibrational free energy for the end-members, especially NaCl is significantly larger than for the mixed systems, Fig. S6. We attribute this to a poor cancellation of errors for systems with few atoms, and thus few phonon branches, despite the seemingly similar agreement in terms of phonon dispersion and DOS. Since the vibrational free energy is calculated from a sum over the phonon frequencies, one can expect mixed systems with more phonon branches to have both overestimated and underestimated frequencies resulting in a cancellation of errors, which is less likely for the pure phases with few atoms."

7. What is the motivation for using the constraints when constructing the CEs? And what are the consequences (in terms of mixing energies and thermodynamic properties) of applying such constraints? For example maybe showing the phase diagram with and without the constraints would help motivate the usage of constraints. Additionally, I think the authors should specify how these constraints are imposed as there are many different ways one can do this.

Using constraints for pure phases is relatively standard practice when constructing cluster expansions, since we are interested in reproducing the mixing (free) energy, which is the relevant property to be sampled in the subsequent Monte Carlo simulation. If no constraints are applied, the pure phases will not have zero mixing energy, which they ideally should. Since we consider the reference (DFT) energies the "ground truth" energies, we believe it is reasonable to constrain our cluster expansion model to reproduce these. However, to show the effect of not constraining to the end-members, we have constructed a cluster expansion model and calculated a phase diagram for (Na/K)Cl without constraints. The results are shown in Fig. S11 and S13, and discussed in the paragraph highlighted in the response to comment 3. Furthermore, we have added the following note on how the constraints are implemented:

"The cluster expansion is fitted to mixing (free) energies, and constrained to reproduce the (free) energies of the end-members exactly with the *get_mixing_energy_constraints* module of ICET"

8. Can the authors spell out more precisely what structure the phonon dispersions are calculated for in Fig 1a,b,c) and Fig3a,b,c), e.g. what are he concentrations, are the structures completely random or ordered etc?

We thank the reviewer for pointing this out, and we have included structural drawings of all the test cases and noted their compositions in Fig. S8 and S17 in the Supporting Information. To clarify, they are not random structures, but selected structures from the training set on which the cluster expansion is constructed. This should be clear now, when the structural drawings are included.

9. Showing the parity plots of e.g. energy and forces for the MLFF would be good for the reader. It is for example hard to evaluate if an error of 4.8meV/Å is small or not, specially since most of the training structures likely have forces close to zero?

We thank the reviewer for this suggestion. We have added parity plots for the forces on the test set between MLFF and DFT for two selected MLFFs in Fig. S5 and S15. We believe that such an out-of-sample error is more meaningful than the error on the training set. Since the energies are weighted very low in the fitting procedure because they are not used for calculating the relevant vibrational properties, we do not benchmark our force field against DFT energies.

10. It would be helpful with some additional details regarding the relaxation process. Why are the structures rattled and then relaxed, and why then symmetrized and then relaxed again? And how is the symmetrization done?

The structures are rattled, since starting from the substituted structures on the parent lattice resulted in a much lower quality MLFF in our initial tests. Starting from rattled structures does require in more relaxation steps (about 50 % more steps here), but it is often used to avoid ending in structures that are local saddle point on the potential energy surface, most strikingly for Jahn-Teller active species. The

symmetrization is performed after the initial ionic convergence, since we often end up in structures very close to a higher symmetry, but the numerical deviations from this symmetry may lead to failure of the DFT code, e.g., if direct and reciprocal lattices are determined to be of different symmetry as a result of the numerical imprecision.

We have added a few sentences on this, including the size of rattling and mentioned that the symmetrization is performed with spglib:

"For each system, all symmetry unique structures with up to 8 metal atoms in the unit cell are included, giving 631 structures for both systems. All relaxations are started from slightly rattled structures using the rattle function of the Atomic Simulation Environment with a standard deviation of 0.05 Å (Larsen et al., 2017). Performing the relaxations without rattling results in too poor sampling of the potential energy surface for training the subsequent MLFF. While the rattling does increase the number of steps in the relaxation trajectories, it also has the benefit of breaking the ideal symmetries to search for lower energy configurations close to the ideal symmetry. The structures are re-symmetrised using spglib (Togo et al., 2018) after convergence of the first ionic relaxation, since small deviation from ideal symmetries after the first relaxation resulted in poor numerical stability. After this, relaxation is restarted as the plane wave basis changes with the size and shape of the simulation cell."

11. It would be useful some more information regarding the phonopy calculations, e.g. how large was the q-point mesh used? Do the authors not include LO-TO splitting in the calculations (is this not relevant for the free energy)?

We have added a few sentences with more information on phonopy parameters. We do not include LO-TO splitting, since determining these would require significant additional calculations for all training data. It is true that it may affect the vibrational free energy, but we expect that the effect is similar on all structures, since the formal charges are the same for K and Na in (Na/K)Cl, and in AgPd, LO-TO splitting does not appear as the system is metallic. We have added the following note on this:

"A 20x20x20 and 40x40x40 q-mesh are used for integrals over the phonon dispersions to calculate DOS and vibrational free energies for $Na_{1-x}K_xCl$ and $Ag_{1-x}Pd_x$, respectively. No LO-TO splitting is included in the models. $Ag_{1-x}Pd_x$ is metallic and thus have no contribution of LOTO splitting. For $Na_{1-x}K_xCl$, LO-TO splitting is relevant, but it requires access to Born effective charges and dielectric constants, which would need to be calculated for each structure, adding a large computational cost. Since the formal ionic charges of Na and K are the same, we expect the effect of LO-TO splitting to be similar on all structures, and thus we neglect it in the present study."

12. The term "end-members" was introduced without any explanation.

We have written that by end-members we mean the pure phases at first instance to make it clear that this is what we mean. We thank the referee for noting this.

13. A suggestion is to also cite sklearn and possibly sources therein for the implementation of ARDR.

We thank the referee for suggesting this and have added a reference to scikit-learn as the implementation of ARDR used by ICET.
